# Prevalence and antibiotic resistance of *Salmonella* isolated from poultry and its environment in the Mekong Delta, Vietnam

**DOI:** 10.14202/vetworld.2021.3216-3223

**Published:** 2021-12-30

**Authors:** Thuan K. Nguyen, Lam T. Nguyen, Trang T. H. Chau, Tam T. Nguyen, Bich N. Tran, Takahide Taniguchi, Hideki Hayashidani, Khai T. L. Ly

**Affiliations:** 1 Department of Veterinary, College of Agriculture, Can Tho University, Campus II, 3/2 Street, Ninh Kieu District, Can Tho City, Vietnam; 2 Cooperative Department of Veterinary Medicine, Division of Animal Life Science, Institute of Agriculture, Tokyo University of Agriculture and Technology, 3-5-8 Saiwai-cho, Fuchu-shi, Tokyo 183-8509, Japan

**Keywords:** antibiotic resistance, chickens, environment, farms, *Salmonella*, wild animals

## Abstract

**Background and Aim:**

*Salmonella* is one of the leading causes of zoonotic and foodborne infectious outbreaks in humans and poultry and its associated environment is a potential reservoir of *Salmonella*. In recent years, the antibiotic resistance of bacteria, including *Salmonella*, has been increasing. This study aimed to investigate the prevalence and antibiotic resistance of *Salmonella* isolated from poultry, its environment, and the pest animals found at poultry farms and households of the Mekong Delta, Vietnam.

**Materials and Methods:**

A total of 3,055 samples were collected from the broiler farms and households of the Mekong Delta from 2017 to 2020. *Salmonella* was isolated using conventional methods (culturing on selective agar – BPLS and biochemical test) and the isolates were examined for antibiotic resistance against 14 antibiotics using the disk diffusion method.

**Results:**

*Salmonella* was isolated from 181 samples (5.92%), which included chicken feces (7.67%), pest animals (5.98%), and environmental samples (4.33%). The environmental samples comprised bedding (5.88%), feed (5.48%), and drinking water (0.70%). The prevalence of *Salmonella* was the highest in rats (15.63%) and geckos (12.25%) followed by ants (2.83%) and cockroaches (2.44%); however, *Salmonella* was not isolated from any fly species. Most of the isolates exhibited resistance to 1-9 antibiotics. The isolates were relatively resistant to chloramphenicol (62.98%), tetracycline (55.80%), ampicillin (54.14%), and sulfamethoxazole/trimethoprim (53.04%). Sixty-two multiple resistance patterns were found in the isolates, with ampicillin-cefuroxime-chloramphenicol-tetracycline- sulfamethoxazole/trimethoprim being the most frequent (7.18%).

**Conclusion:**

The chickens, husbandry environment, and pest animals at poultry farms and households were found to be important *Salmonella* sources in the Mekong Delta. *Salmonella* isolates from these sources also exhibited a wide-ranging resistance to antibiotics as well as several resistance patterns. Hence, biosecurity should be addressed in poultry farms and households to prevent cross-contamination and reduce the spread of *Salmonella* infections.

## Introduction

*Salmonella enterica* subsp. *enterica* is an important infection causing pathogen for warm-blooded animals and commensal organisms for cool-blooded animals, such as reptiles and amphibians [1]. Human salmonellosis is a zoonotic infection as it is caused due to contact with farm or pest animals, either directly through feces or indirectly through food and the environment [2]. Interactions among the chickens, husbandry environment (such as feed, drinking water, and workers), and pest animals (such as rodents, reptiles, and insects) increase the spread of *Salmonella*.

Poultry is known to harbor significant numbers of *Salmonella* serovars without showing clinical signs of the infection [3]. *Salmonella* prevalence is relatively high in chickens and, consequently, farm environments [4-6]. On the other hand, the infected flocks without previous vaccination could be result in almost 100% morbidity and about 20% mortality [7]. Horizontal and vertical transmission by multiple routes complicates the epidemiology of *Salmonella* infections. Moreover, 46.4% of human *Salmonella* infections are linked to animal sources, mostly from poultry [8]. Most foodborne outbreaks in humans due to consumption of contaminated poultry products are also caused due to *Salmonella* [9]. These outbreaks highlight the risk of *Salmonella* infections that originate from poultry and adversely affect human health. However, to the best of our knowledge, the prevalence of *Salmonella* in poultry farms and households of the Mekong Delta is largely unknown.

Due to the overuse of antimicrobial agents, antibiotic-resistant *Salmonella* isolates are found worldwide [10,11]. The misuse of antimicrobials in animal feeds or for the treatment of both humans and animals leads to the selection of resistant bacterial strains. Animals with antibiotic-resistant zoonotic *Salmonella* strains transmit the infection to humans through the food chain [12]. *Salmonella* strains isolated from poultry farms also show multiple resistance patterns for numerous antibiotics [13-17] and display genetic relationships with the *Salmonella* strains detected in humans [14,15]. Thus, the antibiotic-resistant *Salmonella* strains in poultry and farm environments should be carefully monitored.

Some information on the prevalence of *Salmonella* in poultry farms outside of Vietnam is available, but, to the best of our knowledge, a comprehensive study of the sources of *Salmonella* in farms and households of the Mekong Delta has not yet been conducted. Poultry and its environment could be critical sources of *Salmonella* infections for animals and humans in this area.

This study aimed to investigate the prevalence and antibiotic resistance of *Salmonella* isolated from poultry, its environment, and the pest animals found at poultry farms and households of the Mekong Delta, Vietnam.

## Materials and Methods

### Ethical approval

The feces were collected from healthy broilers. Pest animals were caught and dissected in the welfare conditions. This study was carried out under the permission of the laboratory biosafety guideline from Can Tho University, Vietnam.

### Study period, location, and sample collection

From October 2017 to December 2020, 3055 samples were collected from the broiler farms and households of the Mekong Delta, Vietnam. The samples included chicken feces (n=417); environmental samples, including bedding (n=170), feed (n=219), and drinking water (n=142); and pest animals, including rats (n=32), geckos (n=808), flies (n=450), ants (n=530), and cockroaches (n=287).

Chicken feces were collected by swabbing the cloacal into the transporter medium, Carry-Blair (Merck^®^, Germany). The environmental samples, including the feed (250 g), drinking water (1000 mL), and bedding (250 g), were collected directly from the chicken barns, placed in sterilized bags, and stored at 2-8°C. The pest animals (geckos, ants, cockroaches, and rats) were captured in traps and placed separately in sterilized plastic boxes with ventilation holes. All samples were transported to the laboratory on the day of sampling itself.

In the laboratory, the geckos were euthanized by freezing at −20°C for 5 min [18,19] and the rats were euthanized with chloroform (Merck). The animals were dissected at room temperature (28-30°C) and the feces were individually and aseptically collected from the rectum. Ungureanu method for dissecting insects [20] was modified to dissect the collected ants, cockroaches, and flies by freezing at −20°C for 5 min. *Salmonella* isolates were cultured from all the animal bodies. The procedures for animal dissection and feces collection followed the laboratory biosafety guidelines of Can Tho University.

### *Salmonella* isolation and identification

The samples (chicken feces, environmental samples, and pest animal contents) were diluted to 9 times their volume with buffered peptone water (BPW) (Merck^®^, Germany) for incubation at 37°C for 24 h. One milliliter of the BPW broth was transferred into 9 mL of the enrichment broth (Rappaport Vassiliadis soya [RVS] broth) (Merck^®^) for additional incubation at 37°C for 24 h. One loop of the RVS broth was streaked on Brilliant-green Phenol-red Lactose Sucrose agar (Merck^®^) to isolate *Salmonella*. The suspicious *Salmonella* colonies were picked after incubation at 37°C for 24 h. Subsequent biochemical identification was performed as previously described by Tran *et al*. [21].

### Antimicrobial susceptibility testing

A total of 181 *Salmonella* isolates were examined for antibiotic susceptibility with 14 antibiotic agents (Nam Khoa Ltd., Vietnam), including ampicillin (10 μg), amikacin (30 μg), amoxicillin/clavulanic acid (20/10 μg), ceftazidime (30 μg), chloramphenicol (30 μg), colistin (10 μg), cefuroxime (30 μg), doxycycline (30 μg), gentamicin (10 μg), levofloxacin (5 μg), ofloxacin (5 μg), tetracycline (30 μg), streptomycin (10 μg), and sulfamethoxazole/trimethoprim (Bactrim) (23.75/1.25 μg). These antibiotics were used to treat salmonellosis in chickens in the poultry farms of the Mekong Delta. *Escherichia coli* ATCC 25922 and *E. coli* ATCC 35218 were used as controls. The disk diffusion method from the standards of the Clinical Laboratory Standards Institute procedure M02-M07 [22] was conducted to assess the antibiotic resistance of *Salmonella* isolates.

### Statistical analysis

Data were expressed as percentages. The differences in the prevalence of *Salmonella* in the samples collected from the husbandry environment and pest animals were tested using Chi-square test, as were each kind of sample from the farms and households. The statistical significance level was set at p<0.05. Data collection and manipulation were conducted using Microsoft Excel (Microsoft, USA). Statistical analysis was performed using the Statistical Package for the Social Sciences statistical package, version 7.1 (IBM, USA).

## Results

*Salmonella* was detected in 181 of the 3055 samples (5.92%) (Table-1). *Salmonella* was present in the chicken feces, environmental samples, and pest animals (7.67%, 5.98%, and 4.33%, respectively). No statistical difference was observed in the prevalence of *Salmonella* among these samples (p>0.05). There were no significant differences in the comparative prevalence of *Salmonella* in each kind of sample from the farms and households (p>0.05).

**Table 1:** Prevalence of *Salmonella* in the poultry farms and households in the Mekong Delta, Vietnam.

Samples	Households		Farms		Total	
		
	No. of examined samples	No. of positive samples (%)	No. of examined samples	No. of positive samples (%)	No. of examined samples	No. of positive samples (%)
Feces	247	18 (7.29)	170	14 (8.24)	417	32 (7.67)
Environment	336	15 (4.46)	195	8 (4.10)	531	23 (4.33)
Wild animals	1,007	60 (5.96)	1,100	66 (6.00)	2,107	126 (5.98)
Total	1,590	93 (5.85)	1,465	88 (6.01)	3,055	181 (5.92)

*Salmonella* was significantly higher in bedding (5.88%) and feed (5.48%) than in drinking water (0.70%) (p<0.05). No significant difference in the comparative prevalence of *Salmonella* in the bedding and feed samples from the farms and households was observed (p>0.05). However, *Salmonella* was only detected from one drinking water sample from a household (Table-2).

**Table 2:** Prevalence of *Salmonella* in the environmental samples in the poultry farms and households.

Samples	Households		Farms		Total	
		
	No. of examined samples	No. of positive samples (%)	No. of examined samples	No. of positive samples (%)	No. of examined samples	No. of positive samples (%)
Bedding	102	6 (5.88)	68	4 (5.88)	170	10 (5.88)^a^
Feed	137	8 (5.84)	82	4 (4.88)	219	12 (5.48)^a^
Drinking water	97	1 (1.03)	45	0 (0.00)	142	1 (0.70)^b^

The different exponent letters in one column indicate the significant statistical difference (p<0.05)

The prevalence of *Salmonella* in rats (15.63%) and geckos (12.25%) was significantly higher than in ants (2.83%) and cockroaches (2.44%) (p<0.05). However, no significant difference in the comparative prevalence of *Salmonella* in these samples from the farms and households was seen (p>0.05). *Salmonella* was not isolated from any fly or cockroach samples collected from the farms or households (Table-3).

**Table 3:** Prevalence of *Salmonella* in wild animals in the poultry farms and households.

Species	Households		Farms		Total	
		
	No. of examined samples	No. of positive samples (%)	No. of examined samples	No. of positive samples (%)	No. of examined samples	No. of positive samples (%)
Geckos	398	50 (12.56)	410	49 (11.95)	808	99 (12.25)^a^
Ants	260	5 (1.92)	270	10 (3.70)	530	15 (2.83)^b^
Flies	180	0 (0.00)	270	0 (0.00)	450	0 (0.00)
Cockroaches	137	0 (0.00)	150	7 (4.67)	287	7 (2.44)^b^
Rats	32	5 (15.63)	0	0 (0.00)	32	5 (15.63)^a^

The different exponent letters in one column indicate the significant statistical difference (p<0.05)

*Salmonella* isolates were moderately resistant to four antibiotics, chloramphenicol (62.98%), tetracycline (55.80%), ampicillin (54.14%), and sulfamethoxazole/trimethoprim (53.04%). However, the isolates were sensitive (60.22-99.45%) to nine other antibiotics (Table-4).

**Table 4:** The antibiotic resistance of *Salmonella* isolated in the poultry farms and households (n=181).

Antibiotic	Sensitivity		Resistance	
	
	No. of isolates	Percentages	No. of isolates	Percentages
Am	83	45.86	98	54.14
Ac	175	96.69	6	3.31
Ak	180	99.45	1	0.55
Cz	179	98.90	2	1.10
Cu	109	60.22	72	39.78
Cl	67	37.02	114	62.98
Co	159	87.85	22	12.15
Dx	159	87.85	22	12.15
Ge	169	93.37	12	6.63
Lv	164	90.61	17	9.39
Of	176	97.24	5	2.76
Sm	131	72.38	50	27.62
Te	80	44.20	101	55.80
Bt	85	46.96	96	53.04

Am=Ampicillin, Ac=Amoxicillin/clavulanic acid, Ak=Amikacin, Cz=Ceftazidime, Cu=Cefuroxime, Cl=Chloramphenicol, Co=Colistin, Dx=Doxycycline, Ge=Gentamycin, Lv=Levofloxacin, Of=Ofloxacin, Sm=Streptomycin, Te=Tetracycline, Bt=Sulfamethoxazole/trimethoprim

A total of 121/181 *Salmonella* isolates (66.85%) exhibited resistance to 2-9 antibiotics (Table-5) and 62 multiple resistance patterns were observed. The popular resistance pattern included ampicillin-cefuroxime-chloramphenicol-tetracycline-sulfamethoxazole/trimethoprim (7.18%), ampicillin-cefuroxime-chloramphenicol-levofloxacin-tetracycline-sulfamethoxazole/trimethoprim (4.97%), ampicillin-chloramphenicol-tetracycline-sulfamethoxazole/trimethoprim (4.97%), and chloramphenicol-tetracycline-sulfamethoxazole/trimethoprim (3.31%).

**Table 5:** The multiple antibiotic resistance patterns of *Salmonella* isolated in the poultry farms and households (n=181).

No. of resistant antibiotics	Resistance patterns	No. of patterns	No. of isolates (%)
2	Am-Ac	8	2 (1.10)
	Am-Cl		1 (0.55)
	Am-Co		1 (0.55)
	Cl-Bt		2 (1.10)
	Cl-Co		1 (0.55)
	Cl-Te		1 (0.55)
	Sm-Bt		1 (0.55)
	Sm-Te		2 (1.10)
3	Am-Ac-Co	7	1 (0.55)
	Am-Cl-Bt		1 (0.55)
	Am-Cl-Co		1 (0.55)
	Cl-Co-Bt		1 (0.55)
	Cl-Sm-Bt		1 (0.55)
	Cl-Sm-Te		1 (0.55)
	Cl-Te-Bt		6 (3.31)
4	Am-Cl-Sm-Bt	8	3 (1.66)
	Am-Cl-Sm-Te		3 (1.66)
	Am-Cl-Te-Bt		9 (4.97)
	Am-Cu-Cl-Bt		2 (1.10)
	Am-Cu-Cl-Te		1 (0.55)
	Cl-Lv-Te-Bt		1 (0.55)
	Cl-Sm-Te-Bt		2 (1.10)
	Cu-Cl-Te-Bt		2 (1.10)
5	Am-Ac-Cl-Sm-Bt	11	1 (0.55)
	Am-Cl-Co-Te-Bt		1 (0.55)
	Am-Cl-Ge-Sm-Bt		1 (0.55)
	Am-Cl-Ge-Te-Bt		1 (0.55)
	Am-Cl-Lv-Te-Bt		1 (0.55)
	Am-Cl-Sm-Te-Bt		1 (0.55)
	Am-Cu-Cl-Sm-Te		3 (1.66)
	Am-Cu-Cl-Te-Bt		13 (7.18)
	Am-Cu-Sm-Te-Bt		1 (0.55)
	Cl-Dx-Sm-Te-Bt		1 (0.55)
	Cu-Cl-Sm-Te-Bt		1 (0.55)
6	Am-Ac-Cz-Cu-Co-Bt	10	1 (0.55)
	Am-Cl-Dx-Ge-Sm-Te		1 (0.55)
	Am-Cu-Cl-Co-Dx-Sm		1 (0.55)
	Am-Cu-Cl-Co-Dx-Te		4 (2.21)
	Am-Cu-Cl-Co-Te-Bt		2 (1.10)
	Am-Cu-Cl-Dx-Te-Bt		2 (1.10)
	Am-Cu-Cl-Ge-Te-Bt		1 (0.55)
	Am-Cu-Cl-Lv-Te-Bt		9 (4.97)
	Am-Cu-Cl-Of-Te-Bt		1 (0.55)
	Am-Cu-Cl-Sm-Te-Bt		6 (3.31)
7	Ac-Cu-Cl-Ge-Sm-Te-Bt	12	1 (0.55)
	Am-Ak-Cu-Cl-Dx-Sm-Te		1 (0.55)
	Am-Cu-Cl-Co-Dx-Sm-Te		1 (0.55)
	Am-Cu-Cl-Co-Dx-Te-Bt		1 (0.55)
	Am-Cu-Cl-Co-Lv-Te-Bt		1 (0.55)
	Am-Cu-Cl-Dx-Ge-Sm-Te		1 (0.55)
	Am-Cu-Cl-Dx-Sm-Te-Bt		3 (1.66)
	Am-Cu-Cl-Ge-Lv-Te-Bt		1 (0.55)
	Am-Cu-Cl-Ge-Sm-Te-Bt		1 (0.55)
	Am-Cu-Cl-Gr-Sm-Te-Bt		1 (0.55)
	Am-Cu-Cl-Lv-Sm-Te-Bt		1 (0.55)
	Am-Cu-Cl-Of-Sm-Te-Bt		1 (0.55)
8	Am-Cu-Cl-Co-Dx-Sm-Te-Bt	5	3 (1.66)
	Am-Cu-Cl-Dx-Ge-Sm-Te-Bt		1 (0.55)
	Am-Cu-Cl-Dx-Of-Sm-Te-Bt		1 (0.55)
	Am-Cu-Cl-Ge-Lv-Of-Te-Bt		1 (0.55)
	Am-Cz-Cu-Cl-Dx-Sm-Te-Bt		1 (0.55)
9	Am-Cu-Cl-Ge-Lv-Of-Sm-Te-Bt	1	1 (0.55)
Total		62	121 (66.85)

Am=Ampicillin, Ac=Amoxicillin/clavulanic acid, Ak=Amikacin, Cz=Ceftazidime, Cu=Cefuroxime, Cl=Chloramphenicol, Co=Colistin, Dx=Doxycycline, Ge=Gentamycin, Lv=Levofloxacin, Of=Ofloxacin, Sm=Streptomycin, Te=Tetracycline, Bt=Sulfamethoxazole/trimethoprim

## Discussion

*Salmonella* is an important zoonotic pathogen; its presence in animal food products is a continuous threat of infection to humans [23]. *Salmonella* can be transmitted to chickens through horizontal and vertical routes. The largely asymptomatic infected animals can spread infections across the poultry supply chain. The prevalence of *Salmonella* isolated from chicken feces (7.67%) in this study was similar to the previous reports; Alali *et al*. [24] reported that *Salmonella* was isolated from broiler feces in organic (5.6%) and conventional (38.8%) farms in North Carolina, USA. Further, *Salmonella* was detected in chickens in broiler farms in Japan (7.9%) [5], India (2.5%) [25], and Nepal (10.6%) [26]. The prevalence of *Salmonella* isolated from backyard chickens was 3.5% in Paraguay [27] and 12.7% from free-range chickens in China [15]. *Salmonella* was also detected in chickens in pluck shops (6.1%) in Trinidad [28]. Thus, chickens can be considered a potential source of *Salmonella* infections in the Mekong Delta.

The prevalence of non-typhoidal *Salmonella* can be found in environmental reservoirs, infections from which are challenging to control [29]. No significant differences in the prevalence of *Salmonella* among chicken, environmental, and pest animal samples from farms and households were seen in the present study. *Salmonella* might be present regardless of the livestock scale because of continuous cross-contamination of the pathogen among the media present in the farm environments. Ahmed *et al*. [30] determined that chickens and poultry environments were important reservoirs of *Salmonella* in Nigeria. In chicken farms, *Salmonella* can be transmitted through feces, vehicles, workers, clothing, footwear, equipment, water, food, garbage, animals, and other factors [3,31]. A large number of chickens in flocks makes farm biosecurity and good farm management practices difficult [32].

Our field observations indicated that sanitation was not guaranteed in either farms or households. The feeding and drinking water trough use was manual and the chickens could drop their feces into either food or water that could lead to contamination with *Salmonella*. However, we did not detect *Salmonella* in the drinking water samples taken from the farms. Clean water was a concern at the farms and, hence, was replaced more frequently during the day than in the households. Alali *et al*. [24] suggested that the contamination of feed in the feed pans was likely due to fecal droppings. Feeds, especially in the deep litter management systems, can become a potential source of *Salmonella* contamination [33,34]. Further, the prevalence of *Salmonella* was reported as 0.26-38.8% in the feed and 0.6-27.5% in the drinking water [4,24,26,35]. Thus, *Salmonella* can be transmitted back and forth among the chickens through contaminated feed and water. Further investigation could help clarify the cross-contamination routes of *Salmonella* in the environment and flocks at both the farms and households.

The sources of *Salmonella* in poultry might also include pest animals [36]. These animals (rodents, insects, reptiles, etc.) enter the poultry farms and households through holes in the walls or floors. The prevalence of such pest animals poses a threat for the spread of *Salmonella* contamination. Mian *et al*. [37] indicated that flies (*Musca domestica*) were present in large numbers at poultry farms and harbored *Salmonella*. However, *Salmonella* was not isolated from the fly samples in this study. The natural habitats of flies in the Mekong Delta, such as food sources, the frequency of the contact of the flies with *Salmonella* sources (feces, wastes, dead animals, etc.), and hygiene practices in the area might have played a limiting role in the transmission of the pathogen at the farms through flies. The prevalence of *Salmonella* in flies is also related to the seasonal increases in fly activity on the farms and the concurrent cross-contamination within the farm environment [38].

Rodents and lizards might also be important sources of *Salmonella* infections in poultry [35,36]. We isolated *Salmonella* from lizard (2.5%) and rodent (2.8%) sampled from the poultry farms [35]. Nwachukwu *et al*. [39] reported that 25.7% of common house geckos (*Hemidactylus frenatus*) living in Nigeria were *Salmonella* positive. Nguyen *et al*. [40,41] found that the feces of pest geckos harbored *Salmonella* in large numbers and were a potential source of *Salmonella* infections in the Mekong Delta. The incidence of *Salmonella* in ants and cockroaches was relatively low in this study, but these insects remain potential carriers of the pathogen at farms and households. Insects also play an important role in the transmission and spread of various avian pathogens, including *Salmonella*, in the broiler breeder houses [42,43].

The control of pest animals to prevent cross-contamination with *Salmonella* at farms and households in the Mekong Delta is crucial for the management of infections.

Antibiotic resistance may arise due to the indiscriminate use of antimicrobials and their use as growth promoters and chemotherapeutic agents to control diseases at farms [30]. The *Salmonella* isolates from the poultry farms were resistant to 2-8 antibiotics and had 17-25 resistance patterns [13,24,25,44]. Most *Salmonella* isolates in this study showed relative resistance (53.04-62.98%) to four antibiotics (ampicillin, chloramphenicol, tetracycline, and sulfamethoxazole/trimethoprim), which have been used to treat salmonellosis since many years in Vietnam. The use of antibiotics over long periods thus favors the selection of resistant bacterial strains. *Salmonella* isolates in this study also showed 62 resistance patterns. This diversity suggests that *Salmonella* isolates were exposed to various antibiotic agents from multiple sources. Particularly, ampicillin resistance was present in most of the resistance patterns observed in this study, from which we supposed that *Salmonella* isolates from the poultry and husbandry environments have been exposed to significant amounts of ampicillin. Scur *et al*. [13] reported that the highest levels of antimicrobial resistance in *Salmonella* are toward the antimicrobials used over longer periods, which favor the selection of resistant strains. The resistance toward frequently used antimicrobials varies according to geographical locations, production practices, and antimicrobial usage patterns [25]. *Salmonella* isolated from poultry farms show critically high resistance to ampicillin (100%), amoxicillin (99%), and tetracycline (98%) in Bangladesh [44]. *Salmonella* isolated from poultry samples also show high resistance to colistin, ciprofloxacin, doxytetracycline, kanamycin, streptomycin, sulfamethoxazole, and tetracycline (42.1-97.6%) [5,6,14-17,25]. The resistance patterns commonly observed in *Salmonella* isolates from poultry include amoxicillin-ampicillin-ciprofloxacin-erythromycin-gentamycin-penicillin-sulfamethaxole-tetracycline (26.4%) and ampicillin-streptomycin-amoxicillin/clavulanic acid-cephalothin-ceftiofur-cefoxitin (39.7%) [24,44]. Resistance to multiple antibiotics makes the treatment of infections caused by pathogenic bacteria, including *Salmonella*, difficult in both poultry and humans. Thus, the use of antibiotics at poultry farms and households should be controlled to prevent the creation of pathogenic strains resistant to multiple antibiotics, especially *Salmonella*. Further studies should assess the genetic determinants of resistance and their relationship to *Salmonella* isolates from poultry and their environment.

## Conclusion

Samples of chickens, farm environments, and pest animals from poultry farms and households were assessed as potential sources of *Salmonella* in the Mekong Delta, Vietnam. Cross-contamination among these factors may be critical in the spread of *Salmonella* in these environments. The *Salmonella* isolates exhibited resistance against various antibiotics and displayed diverse resistance patterns. The antibiotic-resistant strains of the pathogen pose a risk to animal and human health in this area; therefore, biosecurity should be appropriately managed to prevent cross-contamination and subsequent spread of *Salmonella*. Prospective studies are required to identify the serovars and genetic characteristics of *Salmonella* isolates to further clarify their pathogenicity for poultry and humans.

## Authors’ Contributions

TKN and KTLL: Study conception and design. LTN, TTHC, TTN, and BNT: Acquisition and analysis of data. TKN and LTN: Drafting of the manuscript. TT, HH, and KTLL: Supervised the study. All authors read and approved the final manuscript.
